# Geo–economic variations in epidemiology, ventilation management and outcome of patients receiving intraoperative ventilation during general anesthesia– posthoc analysis of an observational study in 29 countries

**DOI:** 10.1186/s12871-021-01560-x

**Published:** 2022-01-07

**Authors:** Liselotte Hol, Sunny G. L. H. Nijbroek, Ary Serpa Neto, Sabrine N. T. Hemmes, Goran Hedenstierna, Michael Hiesmayr, Markus W. Hollmann, Gary H. Mills, Marcos F. Vidal Melo, Christian Putensen, Werner Schmid, Paolo Severgnini, Hermann Wrigge, Marcelo Gama de Abreu, Paolo Pelosi, Marcus J. Schultz, for the LAS VEGAS study–investigators

**Affiliations:** 1 Department of Anesthesiology, Amsterdam UMC, location AMC, Meibergdreef 9, 1105 AZ Amsterdam, The Netherlands; 2 Department of Intensive Care, Amsterdam UMC, location AMC, Amsterdam, The Netherlands; 3 Department of Critical Care Medicine, Australian and New Zealand Intensive Care Research Centre (ANZIC-RC), Monash University, Melbourne, Australia; 4 Department of Medical Sciences, Clinical Physiology, Uppsala University, Uppsala, Sweden; 5 Division Cardiac, Thoracic, Vascular Anesthesia and Intensive Care, Medical University Vienna, Vienna, Austria; 6 Operating Services, Critical Care and Anaesthesia, Sheffield Teaching Hospitals, Sheffield and University of Sheffield, Sheffield, UK; 7 Department of Anesthesia, Critical Care and Pain Medicine, Massachusetts General Hospital, Boston, MA USA; 8 Department of Anesthesiology and Intensive Care Medicine, University Hospital Bonn, Bonn, Germany; 9 Department of Biotechnology and Life, ASST Sette Laghi Ospedale di Circolo e Fondazio Macchi, University of Insubria, Varese, Italy; 10 Department of Anaesthesiology, Intensive Care Medicine and Emergency Medicine, Pain Therapy, Bermannstrost Hospital Halle, Halle, Germany; 11 Department of Anesthesiology and Intensive Care Medicine, Pulmonary Engineering Group, University Hospital Carl Gustav Carus, Technical University Dresden, Dresden, Germany; 12 Department of Intensive Care and Resuscitation, Cleveland Clinic, Cleveland, OH USA; 13 Department of Outcomes Research, Cleveland Clinic, Cleveland, OH USA; 14 Department of Surgical Sciences and Integrated Diagnostics, Università degli Studi di Genova, Genova, Italy; 15 Anesthesia and Critical Care, IRCCS for Oncology and Neurosciences, San Martino Policlinico Hospital, Genova, Italy; 16 Mahidol–Oxford Tropical Medicine Research Unit (MORU), Mahidol University, Bangkok, Thailand; 17 Nuffield Department of Medicine, University of Oxford, Oxford, UK

**Keywords:** Geo–economic variation, Intraoperative ventilation, ARISCAT score, Postoperative pulmonary complications, ventilation, intraoperative ventilation, Ventilator management

## Abstract

**Background:**

The aim of this analysis is to determine geo–economic variations in epidemiology, ventilator settings and outcome in patients receiving general anesthesia for surgery.

**Methods:**

Posthoc analysis of a worldwide study in 29 countries. Lower and upper middle–income countries (LMIC and UMIC), and high–income countries (HIC) were compared. The coprimary endpoint was the risk for and incidence of postoperative pulmonary complications (PPC); secondary endpoints were intraoperative ventilator settings, intraoperative complications, hospital stay and mortality.

**Results:**

Of 9864 patients, 4% originated from LMIC, 11% from UMIC and 85% from HIC. The ARISCAT score was 17.5 [15.0–26.0] in LMIC, 16.0 [3.0–27.0] in UMIC and 15.0 [3.0–26.0] in HIC (*P* = .003). The incidence of PPC was 9.0% in LMIC, 3.2% in UMIC and 2.5% in HIC *(P < .*001). Median tidal volume in ml kg^− 1^ predicted bodyweight (PBW) was 8.6 [7.7–9.7] in LMIC, 8.4 [7.6–9.5] in UMIC and 8.1 [7.2–9.1] in HIC (*P* < .001). Median positive end–expiratory pressure in cmH_2_O was 3.3 [2.0–5.0]) in LMIC, 4.0 [3.0–5.0] in UMIC and 5.0 [3.0–5.0] in HIC (*P* < .001). Median driving pressure in cmH_2_O was 14.0 [11.5–18.0] in LMIC, 13.5 [11.0–16.0] in UMIC and 12.0 [10.0–15.0] in HIC (*P* < .001). Median fraction of inspired oxygen in % was 75 [50–80] in LMIC, 50 [50–63] in UMIC and 53 [45–70] in HIC *(P < .*001). Intraoperative complications occurred in 25.9% in LMIC, in 18.7% in UMIC and in 37.1% in HIC (*P* < .001). Hospital mortality was 0.0% in LMIC, 1.3% in UMIC and 0.6% in HIC (*P* = .009).

**Conclusion:**

The risk for and incidence of PPC is higher in LMIC than in UMIC and HIC. Ventilation management could be improved in LMIC and UMIC.

**Trial registration:**

Clinicaltrials.gov, identifier: NCT01601223.

**Supplementary Information:**

The online version contains supplementary material available at 10.1186/s12871-021-01560-x.

## Background

Intraoperative ventilation is often mandatory during surgery, to protect the airways and to guarantee adequate gas exchange for as long as the patient is under general anesthesia. However, positive pressure ventilation, even when applied for a relative short period of time, has the potential to cause lung injury, which could translate into postoperative pulmonary complications (PPC). PPC are morbid and even have an association with mortality [1]. Lung–protective ventilation, including the use of a low tidal volume (V_T_) with appropriate positive end–expiratory pressure (PEEP) resulting in a low driving pressure (ΔP), has been shown to prevent PPC [2].

Previous studies have shown geo–economic variations in ventilator management and outcomes in critically ill intensive care unit (ICU) patients – for instance, the ‘Large observational study to UNderstand the Global Impact of Severe Acute respiratory Failure’ (LUNG SAFE), a study in ICU patients with acute respiratory distress syndrome (ARDS), showed that patients in HIC received lower V_T_ and higher PEEP compared to patients in middle–income countries [3]. The LUNG SAFE study also showed that survival of ARDS patients is better in high income countries (HIC). Similar findings come from studies in ICU patients without ARDS – indeed, the ‘PRactice of VENTilation’ (PRoVENT) studies showed better use of ventilation with a low V_T_ in HIC compared to upper and lower middle–income countries (UMIC and LMIC) [4, 5].

In noncardiac surgical patients, remarkable differences in mortality rates have been reported across European countries [6]. It is imaginable that these differences are, at least in part caused by variations in epidemiology as well as intraoperative ventilation management––the latter could be a consequence of lack of local guidelines, or non–compliance with international guidelines, for whatever reason. Geo–economic variations in standard operating procedures, reimbursements, and also between ethical groups could also influence outcomes. To determine the risk for and incidence of PPC, and to compare intraoperative ventilation management and clinical outcomes in geo–economic regions worldwide, we reassessed the database of the conveniently–sized worldwide ‘Local AsSessment of VEntilatory management during General Anaesthesia for Surgery’ (LAS VEGAS) study [7]. We hypothesized that the risk for and actual incidence of PPC differ between LMIC, UMIC and HIC.

## Methods

### Study design

This is a posthoc analysis of the LAS VEGAS study, a prospective 1–week observational study in 146 hospitals across 29 countries, aiming at determining the risk for and actual incidence of PPC and to compare intraoperative ventilation strategies [7]. Both the LAS VEGAS study and this posthoc analysis were carried out in accordance with the recommendations of the ‘STrengthening the Reporting of OBservational studies in Epidemiology’ (STROBE) statement (http://www.strobe-statement.org/). The study protocol was first approved on 22 August 2012 by the institutional review board of the Amsterdam UMC, location AMC, Amsterdam, the Netherlands (W12_190#12.17.0227, chairperson Prof. M.P.M. Burger); each study site sought for local approval to implement the study protocol thereafter. If required, written informed consent was obtained. Surgical patients were enrolled over a predefined period of 1 week, between 14 January and 4 March 2013. The study was registered at clinicaltrials.gov (study identifier NCT01601223).

Adult patients requiring intraoperative ventilation during general anesthesia for surgery were eligible for participation. Patients scheduled for pregnancy–related surgery, surgical procedures outside the operating room, and procedures involving cardiopulmonary bypass were excluded. Patients who had received invasive ventilation in the previous 30 days and patients scheduled to receive thoracic surgery or one–lung ventilation were excluded from participation.

### Data collected in the LAS VEGAS study

Baseline characteristics, ARISCAT risk scores for PPC [8], and details on type of surgery and anesthesia were collected for all patients. Intraoperatively, ventilation parameters, variables, and vital parameters were recorded hourly till the end of surgery. Postoperatively patients were screened daily for occurrence of PPC in the first 5 postoperative days, but was stopped at discharge if this happened before that day. For patients discharged home before postoperative day 5, we assumed they had not developed a PPC after hospital discharge. Of note, some PPC can only be diagnosed e.g., when additional blood sampling or chest imaging is performed –– due to design of the study, these tests were only performed if deemed necessary by the patient’s clinical condition, and this was left to the discretion of the attending doctors. Postoperative day 28 was considered as the end of follow–up.

### Primary endpoint

The coprimary endpoint of this posthoc analysis was the risk for and actual incidence of PPC (as defined below); secondary endpoints were key settings and parameters of intraoperative ventilation, including V_T_, PEEP, ΔP and the fraction of inspired oxygen (FiO_2_). Other secondary endpoints were intraoperative complications (as defined below), hospital stay and all–cause hospital mortality.

### Definitions

We defined the three geo–economic regions using the 2020 World Bank Country Classification system [9].

The ARISCAT risk score was used to calculate the risk for developing PPC, where an ARISCAT risk score of ≥26 points means that a patient has an increased risk for developing one or more PPC (Additional file 2).

Our composite binary endpoint of PPC comprised the following conditions (Additional file 3): respiratory failure (hypoxemia, need for non-invasive positive pressure ventilation, or need for unplanned new or prolonged invasive mechanical ventilation after discharge from the operating room), ARDS (according to the current Berlin definition for ARDS) [10], pneumonia (using clinical and laboratory data), and pneumothorax (observed at the chest radiograph). The PPC, as described above, are all added together and weight equally. Patients who develop at least one PPC were considered as meeting the primary endpoint. PPC can be taken together as they share common pathophysiological pathways [11].

V_T_ per actual bodyweight (ABW) and V_T_ per predicted bodyweight (PBW) were calculated by the following formula: V_T, ABW_ = V_T_/ABW [kg], V_T, PBW_ = V_T_/PBW [kg]. For females, PBW = 45.5 + 0.91 * (height [cm] – 152.4), and for males, PBW = 50.0 + 0.91 * (height [cm] – 152.4). Low V_T_ ventilation was defined as a V_T_ < 8 ml/kg PBW. ΔP was calculated by subtracting PEEP from the plateau pressure.

Intraoperative complications were similar to those used in the parent study and were defined as follows (Additional file 4): any intraoperative desaturation (observed with pulse oximetry), any use of unplanned recruitment maneuvers (RM) (interventions to restore lung aeration), use of ventilator pressure reduction (changes in ventilator settings to decrease the peak or plateau pressure), any new onset of expiratory flow limitation (by visual inspection of the flow curves at the ventilator), hypotension (lasting for 3 min or longer), use of any vasoactive drugs (used to correct hypotension), and any new arrhythmias (as observed at the monitor) [7, 12].

### Statistical analysis plan

No statistical power calculation was conducted for this analysis––instead, the sample size was based on available data. Categorical variables are reported as numbers and relative proportions, continuous variables are reported with median and interquartile range (quartile 25% - quartile 75%). No assumptions for missing data were made. Histograms are used to assess for normality. Depending on data distribution, an ANOVA, Kruskal Wallis test or chi–square test was performed to determine differences among geo–economic regions. If appropriate, a posthoc Dunn test was performed, in which the Bonferroni method was used to adjust for multiplicity. Effect sizes were determined with estimated median differences and Cramér’s V. Length of hospital stay and in–hospital mortality was censored at postoperative day 28.

To adjust for the unequal distribution of effect modifiers on the incidence of PPC, a mixed–effect generalized linear model with binomial distribution was used and results are reported as population–averages. Based on previous literature, ARISCAT, gender, BMI, ASA ≥ 3, functional status, smoking status, COPD, heart failure, malignancy, chronic kidney disease, urgency of surgery, intra-abdominal, intrathoracic, and aortic surgery, and intraoperative Peak Pressure, PEEP, V_T, PBW_, ΔP, need for a blood transfusion, need for vasoactive drugs, and desaturation were considered as clinically relevant possible effect modifiers [13]. Only effect modifiers deemed as clinically relevant and significantly different between groups were added to the model. Centers were introduced as random intercept.

All analyses were conducted in R version 3.5.1 including the packages *lmerTest, stats, tableone, dunn.test, tidyverse, ggplot, lsr,* and *dplyr.* A *P* < .05 was considered statistically significant.

## Results

### Patients

Of 9864 patients included in the current analysis, 405 patients (4%) originated from LMIC, 1076 patients (11%) from UMIC, and 8383 patients (85%) from HIC (Additional file 5). Patient baseline characteristics and anesthesia details are presented in Table 1**,** Additional file 6, and Additional file 7**.** There were no differences in gender distribution, functional status, weight, history of COPD, sleep apnea syndrome, heart failure, malignancy, or chronic kidney disease, and duration of surgery between the geo–economic regions. However, patients from HIC were older than patients from UMIC and LMIC. Of notice, patients from LMIC were median 2 cm taller than patients from UMIC and HIC, 170 [165 to 177] cm in LMIC vs 168 [162 to 175] in UMIC and 168 [162 to 175] cm in HIC (*P* = .015). The proportion of patients with and ASA score ≥ 3 was higher in patients in HIC. Urgent or emergency surgery happened more often in the UMIC and LMIC than in HIC.

**Table 1:** Patient characteristics, in geographic area according the 2020 World Bank Country Classification

	All patients	High income	Upper middle income	Lower middle income	***P***-value (among groups)
**Female (%)**	55.0 (5425/9864)	55.1 (4619/8383)	54.9 (591/1076)	53.1 (215/405)	0.728
**Age, years**	53 [39–66]	54 [40–66]	48 [34–60]	49 [33–62]	< 0.001
**Height, cm**	168 [162–175]	168 [162–175]	168 [162–175]	170 [165–177]	0.015
Height male gender, cm	175 [170–180]	175 [170–181]	175 [170–180]	175 [170–180]	0.084
Height female gender, cm	164 [159–168]	164 [159–168]	163 [159–167]	165 [162–169]	0.003
**Weight, kg**	75 [65–88]	75 [65–88]	75 [65–85]	76 [65–87]	0.623
**Body mass index kg/m**^**2**^	26.2 [23.4–30.0]	26.2 [23.4–30.1]	26.2 [23.4–29.4]	25.7 [23.0–29.4]	0.556
**ASA physical status**					0.017
< 3	78.8 (7756/9840)	78.3 (6549/8361)	81.8 (879/1075)	81.2 (328/404)	
≥ 3	21.7 (2084/9840)	21.7 (1812/8361)	18.2 (196/1075)	18.8 (76/404)	
**Functional status**					0.238
Independent	92.4 (9105/9858)	92.4 (7742/8377)	92.9 (1000/1076)	89.6 (363/405)	
Partially dependent	6.3 (621/9858)	6.3 (526/8377)	5.6 (60/1076)	8.6 (35/405)	
Totally dependent	1.3 (132/9858)	1.3 (109/8377)	1.5 (16/1076)	1.7 (7/405)	
**ARISCAT score**	15.0 [3.0 to 26.0]	15.0 [3.0 to 26.0]	16.0 [3.0 to 27.0]	17.5 [15.0 to 26.0]	0.003
**ARISCAT group**					0.563
Low	75.9 (7147/9413)	76.1 (6128/8053)	74.7 (763/1022)	75.7 (256/338)	
Intermediate	19.2 (1811/9413)	19.0 (1532/8053)	21.0 (215/1022)	18.9 (64/338)	
High	4.8 (455/9413)	4.9 (393/8053)	4.3 (44/1022)	5.3 (18/338)	
Preoperative SpO_2_, %	98 [96, 99]	98 [96, 99]	98 [96, 99]	98 [96, 99]	< 0.001
**Comorbidities**					
COPD	6.0 (596/9864)	6.0 (503/8383)	6.0 (65/1076)	6.9 (28/408)	0.753
Heart failure	5.9 (585/9864)	5.8 (486/8383)	6.3 (68/1076)	7.6 (31/405)	0.257
Obstructive sleep apnoea	2.1 (205/9864)	2.2 (183/8383)	1.6 (17/1076)	1.2 (5/405)	0.204
Metastatic cancer	4.0 (392/9864)	4.1 (347/8383)	3.1 (33/1076)	3.0 (12/405)	0.135
Chronic kidney disease	3.1 (310/9864)	3.3 (276/8383)	2.2 (24/1076)	2.5 (10/405)	0.140
Smoker	23.2 (2290/9864)	22.7 (1906/8383)	27.0 (291/1076)	23.1 (93/405)	0.008
Urgency of surgery*					< 0.001
Elective	88.9 (8765/9862)	90.2 (7557/8381)	83.1 (894/1076)	77.5 (314/405)	
Urgency	8.6 (845/9862)	8.0 (667/8381)	10.3 (111/1076)	16.5 (67/405)	
Emergency	2.6 (252/9862)	1.9 (157/8381)	6.6 (71/1076)	5.9 (24/405)	
**Duration of surgery, minutes**	73 [42, 125]	71 [40, 125]	75 [45, 125]	75 [42, 125]	0.371
**Duration of anesthesia, minutes**	103 [66, 160]	103 [65, 162]	100 [70, 150]	105 [63, 152]	0.546
**Surgical approach**					
Open abdominal	18.0 (1773/9864)	17.7 (1487/8383)	19.1 (205/1076)	20.0 (81/405)	0.318
Laparoscopic abdominal	17.6 (1737/9864)	17.5 (1468/8383)	17.4 (187/1076)	20.2 (82/405)	0.361
Laparoscopic assisted abdominal	1.7 (167/9864)	1.7 (143/8383)	1.8 (19/1076)	1.2 (5/405)	0.758
Peripheral incision	18.5 (1827/9864)	19.5 (1633/8383)	12.2 (131/1076)	15.6 (63/405)	< 0.001
None of the above	44.9 (4427/9864)	44.3 (3714/8383)	49.9 (537/1076)	43.5 (176/405)	0.002
**Type of surgery**					
Lower gastro-intestinal	11.1 (1096/9864)	11.0 (920/8383)	10.2 (110/1076)	16.3 (66/405)	0.002
Upper gastro-intestinal	13.8 (1357/9864)	13.2 (1107/8383)	16.1 (173/1076)	19.0 (77/405)	< 0.001
Peripheral vascular	3.1 (309/9864)	3.1 (261/8383)	3.5 (38/1076)	2.5 (10/405)	0.559
Aortic	0.6 (64/9864)	0.7 (62/8383)	0.2 (2/1076)	0.0 (0/405)	0.026
Neurological, head or neck	20.3 (2006/9864)	19.4 (1627/8383)	27.0 (290/1076)	22.0 (89/405)	< 0.001
Urological or kidney	8.7 (858/9864)	8.8 (741/8383)	8.6 (93/1076)	5.9 (24/405)	0.127
Gynecological	11.6 (1141/9864)	11.8 (993/8383)	10.1 (109/1076)	9.6 (39/405)	0.117
Endocrine	2.0 (194/9864)	1.9 (156/8383)	2.8 (30/1076)	2.0 (8/405)	0.119
Transplant	0.3 (34/9864)	0.3 (26/8383)	0.7 (8/1076)	0.0 (0/405)	0.036
Plastic or Cutaneous	10.5 (1037/9864)	11.0 (920/8383)	8.3 (89/1076)	6.9 (28/405)	0.001
Bone or joint	16.2 (1595/9864)	16.9 (1418/8383)	11.6 (125/1076)	12.8 (52/405)	< 0.001
Other procedure	5.9 (585/9864)	6.1 (508/8383)	5.4 (58/1076)	4.7 (19/405)	0.381
Epidural catheter	4.8 (476/9859)	5.3 (420/7958)	3.3 (49/1498)	1.7 (7/403)	< 0.001
Muscle paralysis agents	84.1 (8275/9845)	82.5 (6563/7951)	90.2 (1345/1491)	91.1 (367/403)	< 0.001
Neuromuscular blockade reversal agent	34.0 (3294/9687)	31.3 (2463/7860)	43.8 (640/1461)	52.2 (191/366)	< 0.001
Neuromuscular monitoring	18.1 (1786/9850)	20.8 (1654/7949)	5.4 (81/1498)	12.7 (51/403)	< 0.001
Need for a blood transfusion	3.3 (330/9864)	3.1 (260/8383)	5.0 (54/1076)	4.0 (16/405)	0.005

Data presented as median with interquartile range [25th to 75th quartile] or % (n/total). Total numbers are different because of missing values. Depending on data distribution, an ANOVA, Kruskal Wallis or Chi square test was performed to determine differences among geo–economic regions

^*^ Urgency of surgery: elective: surgery that is scheduled in advance because it does not involve a medical emergency; urgent: surgery required within < 48 h; emergency: non-elective surgery performed when the patient’s life or wellbeing is in direct jeopardy

*ASA* American Society of Anesthesiology, *ARISCAT* Assess Respiratory Risk in Surgical Patients in Catalonia, *SpO*_*2*_ oxyhaemoglobin saturation by pulse oximetry, *COPD* Chronic Obstructive Pulmonary Disease

### The risk for and actual incidence of postoperative pulmonary complications

**Fig. 1:**
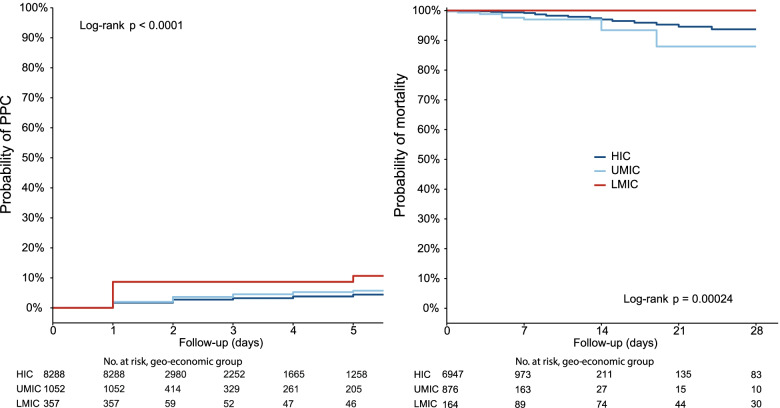
Kaplan–Meier curves for postoperative pulmonary complications and hospital mortality. Patients who were lost to follow–up due to hospital discharge were assumed not to have developed PPC

Data to calculate the ARISCAT risk score was available for 9413 patients. The median ARISCAT risk score was 17.5 [15.0 to 26.0] in LMIC, versus 16.0 [3.0 to 27.0] in UMIC and 15.0 [3.0 to 26.0]) in HIC (*P* = .003). The proportions of patients with a low, an intermediate and a high risk for PPC, however, was not different across the geo–economic regions (Table 1). Data to calculate the incidence of PPC were available in 9697 patients. The incidence of PPC was 9.0% in LMIC, versus 3.2% in UMIC and 2.5% in HIC (*P* < .001) (Table 2, Fig. 1). After adjustment for effect modifiers and compared to the LMIC, the incidence of PPC remained lower in UMIC (OR 0.054 (0.026 to 0.110), *P* < .001) and HIC (OR 0.035 (0.020 to 0.062), *P* < .001) (Additional file 8).

**Table 2:** Postoperative complications, in geographic area according the 2020 World Bank Country Classification

	High income	Upper middle income	Lower middle income	***P***-value (among groups)
PPC	2.5 (204/8288)	3.2 (34/1052)	9.0 (32/357)	< 0.001
Pneumothorax	0.1 (10/8288)	0.3 (3/1052)	0.0 (0/357)	0.304
Respiratory failure	1.3 (108/8288)	1.7 (18/1052)	8.4 (30/357)	< 0.001
Pneumonia	0.4 (30/8288)	1.0 (10/1052)	0.0 (0/357)	0.009
ARDS	0.1 (5/8288)	0.4 (4/1052)	0.0 (0/357)	0.005
Unplanned new invasive mechanical ventilation	1.2 (97/8288)	0.9 (9/1052)	0.3 (1/357)	0.207
Length of hospital stay, days	1 [0 to 4]	2 [0 to 5]	8 [1 to 21]	< 0.001
In-hospital mortality	0.6 (43/7627)	1.3 (13/1017)	0 (0/329)	0.009

Data presented as % (n/total). *RM* Recruitment maneuvers, *PPC* Postoperative Pulmonary Complication, *ARDS* Acute Respiratory Distress Syndrome

### Intraoperative ventilation management

**Fig. 2:**
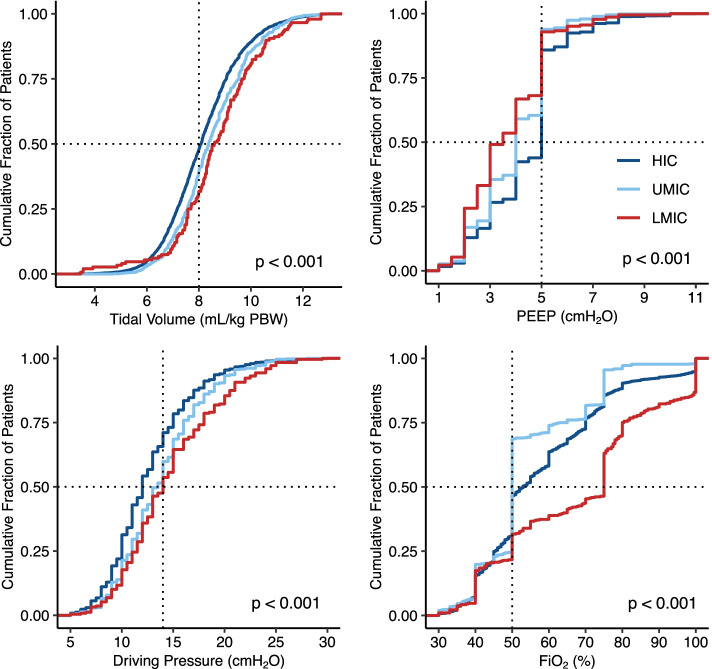
Cumulative distribution plots for the median values of the ventilatory parameters during the intraoperative period and stratified by geo*–*economic group. PBW calculated according to the standard formula. Unadjusted *p*-value comparing multiple groups. Abbreviations: Tidal volume (V_T_), Positive End–expiratory Pressure (PEEP), Driving pressure (ΔP) and Fraction inspired oxygen (FiO_2_) per income group according to the World Bank country classification 2020

Key ventilator variables and parameters are shown in Table 3 and Fig. 2. Median V_T, ABW_ and V_T, PBW_ were higher in LMIC compared to UMIC and HIC (*P* < .001). Median PEEP increased from LMIC to UMIC and HIC (*P* < .001). Median ΔP and FiO_2_ decreased from LMIC to UMIC and HIC (*P* < .001).

**Table 3:** Ventilatory practice, in geographic area according the 2020 World Bank Country Classification

	High income	Upper middle income	Lower middle income	***P***-value (among groups)
Mode of ventilation				< 0.001
Volume controlled	70.0 (5799/8287)	70.8 (734/1037)	72.0 (283/393)	
Pressure controlled	17.9 (1481/8287)	3.3 (34/1037)	14.2 (56/393)	
Pressure support	1.2 (97/8287)	0.3 (3/1037)	1.0 (4/393)	
Spontaneous	5.1 (424/8287)	7.4 (77/1037)	9.4 (37/393)	
Other	5.9 (486/8287)	18.2 (189/1037)	3.3 (13/393)	
Tidal Volume				
Absolute, mL	500 [450 to 550]	500 [480 to 570]	578 [500 to 600]	< 0.001
PBW, ml kg^−1^	8.1 [7.2 to 9.1]	8.4 [7.6 to 9.5]	8.6 [7.7 to 9.7]	< 0.001
ABW, ml kg^−1^	6.7 [5.8 to 7.7]	6.9 [6.1 to 7.8]	7.4 [6.5 to 8.6]	< 0.001
Low V_T_	3513 (47.5)	342 (38.9)	47 (31.5)	< 0.001
PEEP, cmH_2_O	5.0 [3.0 to 5.0]	4.0 [3.0 to 5.0]	3.3 [2.0 to 5.0]	< 0.001
Peak pressure, cmH_2_O	17.5 [15.0 to 21.0]	18.0 [15.0 to 21.0]	20.0 [17.0 to 25.0]	< 0.001
Driving pressure, cmH_2_O	12.0 [10.0 to 15.0]	13.5 [11.0 to 16.0]	14.0 [11.5 to 18.0]	< 0.001
Plateau pressure, cmH_2_O	15.5 [13.0 to 18.0]	16.0 [13.5 to 19.0]	17.0 [14.0 to 20.0]	< 0.001
FiO_2_, %	53.0 [45.0 to 70.0]	50.0 [50.0 to 63.0]	75.0 [50.0 to 80.0]	< 0.001
EtCO_2_, mm Hg	34.0 [31.0 to 36.8]	32.0 [30.0 to 35.0]	32.3 [30.0 to 35.5]	< 0.001
Respiratory rate, rpm	12.0 [11.5 to 13.0]	12.0 [12.0 to 13.0]	12.0 [12.0 to 13.0]	< 0.001
Dynamic lung compliance, ml (cmH_2_O)^−1^	35.2 [28.4 to 43.3]	33.9 [27.8 to 40.4]	31.2 [24.6 to 38.0]	< 0.001

Data presented as median with interquartile range [25th to 75th quartile] or % (n/total)

*ABW* Actual BodyWeight, *EtCO*_*2*_ End tidal Carbon dioxide, *PBW* Predicted BodyWeight; *PEEP* Positive-end-expiratory Pressure; V_T_

### Intraoperative complications, length of stay and mortality

Intraoperative complications occurred more often in HIC and LMIC than in UMIC (*P* < .001) (Table 4). Length of hospital stay was higher in LMIC compared to that in UMIC and HIC (*P* < .001), and all–cause hospital mortality was higher in UMIC than HIC (*P* = .009) (Table 2).

**Table 4:** Intraoperative complications, in geographic area according the 2020 World Bank Country Classification

	High income	Upper middle income	Lower middle income	***P***-value (among groups)
Intraoperative complications	37.1 (3101/8368)	18.7 (201/1076)	25.9 (105/405)	< 0.001
Desaturation	4.0 (334/8364)	4.1 (44/1076)	2.2 (9/404)	0.196
Use of unplanned RM	3.5 (296/8358)	2.4 (26/1076)	2.5 (10/403)	0.094
Use of ventilator pressure reduction	2.9 (243/8352)	2.2 (24/1076)	3.7 (15/402)	0.260
New onset of expiratory flow limitation	0.5 (42/9786)	0.7 (7/1074)	0.7 (3/401)	0.685
Hypotension	28.6 (2395/8365)	13.7 (147/1076)	18.6 (75/404)	< 0.001
Use of vasoactive drugs	24.7 (2067/8364)	8.7 (94/1076)	11.6 (47/404)	< 0.001
Any new arrhythmias	0.5 (45/8359)	0.8 (9/1076)	1.5 (6/403)	0.034

Data presented as % (n/total). *RM: Recruitment maneuvers*

## Discussion

This posthoc analysis of the conveniently sized LAS VEGAS study shows that the risk for and actual incidence of PPC decreases from LMIC to UMIC and HIC. The analysis also shows significant geo–economic differences in ventilation management, as well as in the incidence of intraoperative complications, length of in–hospital stay and mortality.

To our best knowledge, this is the first study examining whether geo–economic variation in the risk for and actual incidence of PPC in surgical patients exist. We used the database of a prospective study that included surgical patients requiring intraoperative ventilation for various types of surgery, that included centers worldwide. The LAS VEGAS study was performed in both community and teaching hospitals, increasing the generalizability of the findings. Another strength is that data were collected within 1 week, preventing against the risk of temporal changes in risks for and incidences of PPC, intraoperative ventilation management and outcomes.

Our analysis rejects the null hypothesis that there are no geo–economic variations in the risk for and incidence of PPC. While the higher incidence of PPC in LMIC might partly be explained by a higher ARISCAT score in patients in these regions, the fraction of patients at an increased or high risk for PPC in LMIC was comparable to UMIC and HIC. The fraction of patients undergoing upper abdominal surgery was higher in LMIC, which is important as especially this type of surgery has a strong association with occurrence of pulmonary complications after surgery [14]. An alternative explanation for the higher incidence of PPC in LMIC could be that lung–protective ventilation was used less often in patients in these regions. Two meta–analyses showed intraoperative ventilation with a high V_T_ or a high ΔP to have an association with the development of PPC [15, 16]. We here show that both V_T_ and ΔP were higher in LMIC compared to that in UMIC and HIC.

In the LAS VEGAS study, we used strict definitions for PPC to minimize regional variations in terminology. Each PPC was easy to score; additional tests were not required by the study protocol and follow–up of PPC ended at patient’s discharge. To ensure accurate data collection, standard operating procedures for data entry were present for all investigators. The Case Report Form of the LAS VEGAS study was developed with the assistance of the European Society of Anesthesiology––Clinical Trial Network, resulting in a straightforward and easy–to–use form. Furthermore, national coordinators were delegated to assist, train and monitor local data collectors [7, 12]. However, still we cannot exclude that there were some regional variations in the process of diagnosing and reporting PPC––for instance, some PPC can only be diagnosed when additional blood samples are taken or if pulmonary imaging is performed. Geo–economic variations in standard operating procedures for diagnostics in the perioperative period could interfere with our findings.

Respiratory failure was the most frequently diagnosed component of PPC in all three geo–economic groups. The incidence of respiratory failure was significantly higher in LMIC compared to its incidence in UMIC and HIC. It is unknown if the occurrence of residual curarization, a possible cause of respiratory failure, differed between the geo–economic groups. Of note, we did find the use of neuromuscular blocking agents and antagonists to be higher in LMIC compared to UMIC and HIC.

Length of hospital stay in LMIC was 4 times higher than in UMIC and even 8 times higher than in HIC. This could, at least in part, be explained by the difference in the incidence of PPC. PPC occurred significantly more often in LMIC compared to UMIC and HIC. Indeed, earlier studies showed the development of PPC to be associated with an increased length of in-hospital stay [1]. Regional variations in guidelines and protocols for hospital discharge may also explain this difference.

Several studies described the development of PPC to be associated with increased mortality [1, 8]. In our study, the incidence of PPC was too low to confirm such an association. However, we did find a higher all–cause hospital mortality rate in UMIC compared to HIC. Our analysis showed a mortality rate of 1.3% in UMIC and 0.6% in HIC, which is lower than the 4% reported in the European Surgical Outcomes study (EuSOS) [6]. In EuSOS, 46,539 patients undergoing noncardiac surgery in 489 hospitals across 28 European nations were included. The differences in mortality between our study and EuSOS could partly be explained by differences in baseline characteristics. In the EuSOS cohort patients were older, and the fraction of patients with ASA ≥3, and with metastatic diseases was slightly higher. These three baseline characteristics are, according to the EuSOS analysis independently associated with mortality. A second possible explanation might be that the follow–up period in the EuSOS cohort was twice as long as in the LAS VEGAS cohort, which could increase the registered incidence of mortality. Our reported incidence of mortality is more comparable with other studies evaluating clinical outcomes in surgical patients [17–20].

Intraoperative complications, specifically hypotension and the use of vasoactive drugs, occurred more often in HIC compared to UMIC and LMIC. Patients from HIC were ventilated with a higher PEEP and received more frequently an epidural catheter than patients from UMIC and LMIC, both known to be risk factors for hypotension [21–23]. It is uncertain if other characteristics, such as depth of anesthesia, play a role herein. Also, important to note is that differences in the availability and use of monitoring and recording systems in the operating rooms between HIC and UMIC and LMIC could explain the differences in intraoperative complications. Last but not least, reporting could have been hampered by higher workloads for anesthesiologists and anesthetic nurses in LMIC and UMIC compared to HIC.

We found small differences in preoperative saturation and intraoperative respiratory rate. These differences reached statistical significance but were probably of no clinical meaning. This interpretation is supported by the between–group comparable median, interquartile ranges, and estimated median differences.

Our study has limitations. One limitation is the unequal distribution of patients between the geo–economic groups. Indeed, the number of patients in HIC was 8 times higher than in UMIC, and even 20 times higher than in LMIC. This increases the risk of type II errors. Furthermore, it is uncertain if the small number of patients in the LMIC gives an adequate representation of this latter geo–economic group. Patients from LMIC were median 2 cm taller compared to patients from UMIC and HIC which is not to be expected. The mortality rate of zero in LMIC was unexpected as well. These findings could be the result of the small group size since another plausible explanation is lacking. We also did not have patients that received surgery in a low–income country, the fourth group of the 2020 World Bank country classifications. One additional limitation is that the LAS VEGAS study was conducted in 2013. Perioperative care is not expected to have been changed dramatically over the last two decades, but is uncertain if our findings are completely generalizable to the present.

It should be stressed that the findings of this posthoc analysis serve as hypothesis–generating evidence. A posthoc analysis has a lower positive predictive value by design, which increases the risk for a type I error [24]. However, multiple analysis performed on various databases show geo–economic variations in ventilation and clinical outcomes, making it more plausible that the null hypothesis is rejected correctly [3–5]. Additional research such as a meta–analysis is required to further establish this matter.

The increased incidence of PPC and the decreased use of lung–protective ventilation in LMIC should concern us. An association between gross national income per capita and clinical outcomes has been found in other cohorts as well. Several studies showed lower income to be associated with worse survival in ICU patients diagnosed with ARDS or with sepsis [3, 25, 26]. The causes of these geo–economic variations in clinical outcomes falls beyond the scope of this analysis and remain uncertain. Additional research is needed to provide us with more insights and possible solutions to reduce the impact of geo-economics on the use of preventive measures and clinical outcomes.

## Conclusion

In this worldwide study of intraoperative ventilation under general anesthesia for surgery, the risk for and actual incidence of PPC was higher in LMIC compared to UMIC and HIC. During intraoperative ventilation, patients in LMIC were ventilated with higher V_T_ and ΔP, higher FiO_2_ but lower PEEP compared to patients from UMIC and HIC. These findings raise the awareness of geo–economic differences in clinical outcome and ventilation management of surgical patients.

## Supplementary Information

**Additional file 1.** Full list of LAS VEGAS collaborators. A list of all LAS VEGAS researchers and their affiliations.

**Additional file 2.** ARISAT score. The ARISCAT risk score is used to calculate the risk for developing postoperative pulmonary complications.

**Additional file 3.** Definitions of postoperative pulmonary complications. A description of the definitions of postoperative pulmonary complications used in this analysis.

**Additional file 4.** Definitions of intraoperative complications. A description of the definitions of intraoperative complications used in this analysis.

**Additional file 5.** CONSORT flow chart of the study population. Flowchart with information on how the study population was obtained.

**Additional file 6.** Posthoc pairwise analysis for numerical data. Posthoc Dunn’s test for pairwise multiple comparison of the ranked data.

**Additional file 7.** Posthoc pairwise analysis for categorical data. Chi-square test for pairwise comparison.

**Additional file 8.** Multivariate model of factors associated with the development of PPC. HIC and UMIC were compared to LMIC and centers were entered as random effect.

## Data Availability

The dataset and code used for this post-hoc analysis are available from the corresponding author upon reasonable request.

## Abbreviations

ABW: Actual Body Weight
ARDS: Acute Respiratory Distress Syndrome
ARISCAT: Assess Respiratory Risk in Surgical Patients in Catalonia
ASA: American Society of Anesthesiology
FiO_2_: Inspired Oxygen Fraction
HIC: High–Income Countries
ICU: Intensive Care Unit
LMIC: Lower Middle–Income Countries
PaO2: Partial Pressure of Oxygen
PEEP: Positive End-Expiratory Pressure
PPC: Postoperative Pulmonary Complications
PBW: Predicted Body Weight
RM: Recruitment Maneuver
SpO_2_: Peripheral Oxygen Saturation
UMIC: Upper Middle–Income Countries
V_T_: Tidal Volume
WBC: White Blood Cell
ΔP: Driving Pressure
