# Supplementary material for: Geo–economic variations in epidemiology, ventilation management and outcome of patients receiving intraoperative ventilation during general anesthesia– posthoc analysis of an observational study in 29 countries
Source: BMC Anesthesiol. 2022 Jan 7;22:15. doi: 10.1186/s12871-021-01560-x (PMC8740416; doi:10.1186/s12871-021-01560-x)
Supplement: Supplementary file 1 — Additional file 1. Full list of LAS VEGAS collaborators. A list of all LAS VEGAS researchers and their affiliations. [file 12871_2021_1560_MOESM1_ESM.docx]

**Additional file 1.** List of LAS VEGAS study Network Collaborators

Austria

***LKH Graz, Graz:*** Wolfgang Kroell, Helfried Metzler, Gerd Struber, Thomas Wegscheider

***AKH Linz, Linz*:** Hans Gombotz

***Medical University Vienna*:** Michael Hiesmayr, Werner Schmid, Bernhard Urbanek

Belgium

***UCL - Cliniques Universitaires Saint Luc Brussels*:** David Kahn, Mona Momeni, Audrey Pospiech, Fernande Lois, Patrice Forget, Irina Grosu

***Universitary Hospital Brussels (UZ Brussel)*:** Jan Poelaert, Veerle van Mossevelde, Marie-Claire van Malderen

***Het Ziekenhuis Oost Limburg (ZOL), Genk*:** Dimitri Dylst, Jeroen van Melkebeek, Maud Beran

***Ghent University Hospital, Gent*:** Stefan de Hert, Luc De Baerdemaeker, Bjorn Heyse, Jurgen Van Limmen, Piet Wyffels, Tom Jacobs, Nathalie Roels, Ann De Bruyne

***Maria Middelares, Gent*:** Stijn van de Velde

***European Society of Anaesthesiology, Brussels:*** *Brigitte Leva, Sandrine Damster, Benoit Plichon*

Bosnia and Herzegovina

***General Hospital “prim Dr Abdulah Nakas” Sarajevo*:** Marina Juros-Zovko, Dejana  Djonoviċ- Omanoviċ

Croatia

***General Hospital Cakovec, Cakovec*:** Selma Pernar

***General Hospital Karlovac, Karlovac*:** Josip Zunic, Petar Miskovic, Antonio Zilic

***University Clinical Hospital Osijek, Osijek*:** Slavica Kvolik, Dubravka Ivic, Darija Azenic-Venzera, Sonja Skiljic, Hrvoje Vinkovic, Ivana Oputric

***University Hospital Rijeka, Rijeka*:** Kazimir Juricic, Vedran Frkovic

***General Hospital Dr J Bencevic, Slavonski Brod*:** Jasminka Kopic, Ivan Mirkovic

***University Hospital Center Split, Split*:** Nenad Karanovic, Mladen Carev, Natasa Dropulic

***University Hospital Merkur, Zagreb*:** Jadranka Pavicic Saric, Gorjana Erceg, Matea Bogdanovic Dvorscak

***University Hospital Sveti Duh, Zagreb*:** Branka Mazul-Sunko, Anna Marija Pavicic, Tanja Goranovic

***University Hospital, Medical school, “Sestre milosrdnice” (Sister of Charity), Zagreb*:** Branka Maldini, Tomislav Radocaj, Zeljka Gavranovic, Inga Mladic-Batinica, Mirna Sehovic

Czech Republic

***University Hospital Brno, Brno*:** Petr Stourac, Hana Harazim, Olga Smekalova, Martina Kosinova, Tomas Kolacek, Kamil Hudacek, Michal Drab

***University Hospital Hradec Kralove, Hradec Kralove*:** Jan Brujevic, Katerina Vitkova, Katerina Jirmanova

***University Hospital Ostrava, Ostrava*:** Ivana Volfova, Paula Dzurnakova, Katarina Liskova

***Nemocnice Znojmo, Znojmo*:** Radovan Dudas, Radek Filipsky

Egypt

***El Sahel Teaching hospital, Cairo*:** Samir el Kafrawy

***Kasr Al-Ainy Medical School, Cairo University*:** Hisham Hosny Abdelwahab, Tarek Metwally, Ahmed Abdel-Razek

***Beni Sueif University Hospital, Giza*:** Ahmed Mostafa El-Shaarawy, Wael Fathy Hasan, Ahmed Gouda Ahmed

***Fayoum University Hospital, Giza*:** Hany Yassin, Mohamed Magdy, Mahdy Abdelhady

***Suis medical Insurance Hospital, Suis*:** Mohamed Mahran

Estonia

***North Estonia Medical Center, Tallinn*:** Eiko Herodes, Peeter Kivik, Juri Oganjan, Annika Aun

***Tartu University Hospital, Tartu*:** Alar Sormus, Kaili Sarapuu, Merilin Mall, Juri Karjagin

France

***University Hospital of Clermont-Ferrand, Clermont-Ferrand*:** Emmanuel Futier, Antoine Petit, Adeline Gerard

***Institut Hospitalier Franco-Britannique, Levallois-Perret*:** Emmanuel Marret, Marc Solier

***Saint Eloi University Hospital, Montpellier*:** Samir Jaber, Albert Prades

Germany

***Fachkrankenhaus Coswig, Coswig*:** Jens Krassler, Simone Merzky

***University Hospital Carl Gustav Carus, Dresden*:** Marcel Gama de Abreu, Christopher Uhlig, Thomas Kiss, Anette Bundy, Thomas Bluth, Andreas Gueldner, Peter Spieth, Martin Scharffenberg, Denny Tran Thiem, Thea Koch

***Duesseldorf University Hospital, Heinrich-Heine University*:** Tanja Treschan, Maximilian Schaefer, Bea Bastin, Johann Geib, Martin Weiss, Peter Kienbaum, Benedikt Pannen

***Diakoniekrankenhaus Friederikenstift, Hannover*:** Andre Gottschalk, Mirja Konrad, Diana Westerheide, Ben Schwerdtfeger

***University of Leipzig, Leipzig*:** Hermann Wrigge, Philipp Simon, Andreas Reske, Christian Nestler

Greece

***“Alexandra” general hospital of Athens, Athens*:** Dimitrios Valsamidis, Konstantinos Stroumpoulis

***General air force hospital, Athens*:** Georgios Antholopoulos, Antonis Andreou, Dimitris Karapanos

***Aretaieion University Hospital, Athens*:** Kassiani Theodoraki, Georgios Gkiokas, Marios-Konstantinos Tasoulis

***Attikon University Hospital, Athens*:** Tatiana Sidiropoulou, Foteini Zafeiropoulou, Panagiota Florou, Aggeliki Pandazi

***Ahepa University Hospital Thessaloniki, Thessaloniki*:** Georgia Tsaousi, Christos Nouris, Chryssa Pourzitaki,

Israel

***The Lady Davis Carmel Medical Center, Haifa*:** Dmitri Bystritski, Reuven Pizov, Arieh Eden

Italy

***Ospedale San. Paolo Bari, Bari*:** Caterina Valeria Pesce, Annamaria Campanile, Antonella Marrella

***University of Bari “Aldo Moro”, Bari*:** Salvatore Grasso, Michele De Michele

***Institute for Cancer Research and treatment, Candiolo, Turin*:** Francesco Bona, Gianmarco Giacoletto, Elena Sardo

***Azienda Ospedaliera per l’emergenza Cannizzaro, Catania*:** Luigi Giancarlo Vicari Sottosanti

***Ospedale Melegnano, Cernuso, Milano*:** Maurizio Solca

***Azienda Ospedaliera – Universitaria Sant’Anna, Ferrara*:** Carlo Alberto Volta, Savino Spadaro, Marco Verri, Riccardo Ragazzi, Roberto Zoppellari

***Ospedali Riuniti Di Foggia - University of Foggia, Foggia*:** Gilda Cinnella, Pasquale Raimondo, Daniela La Bella, Lucia Mirabella, Davide D'antini

***IRCCS AOU San Martino IST Hospital, University of Genoa, Genoa*:** Paolo Pelosi, Alexandre Molin, Iole Brunetti, Angelo Gratarola, Giulia Pellerano, Rosanna Sileo, Stefano Pezzatto, Luca Montagnani

***IRCCS San Raffaele Scientific Institute, Milano*:** Laura Pasin, Giovanni Landoni, Alberto Zangrillo, Luigi Beretta, Ambra Licia Di Parma, Valentina Tarzia, Roberto Dossi, Marta Eugenia Sassone

***Istituto europeo di oncologia – ieo, Milano*:** Daniele Sances, Stefano Tredici, Gianluca Spano, Gianluca Castellani, Luigi Delunas, Sopio Peradze, Marco Venturino

***Ospedale Niguarda Ca'Granda Milano, Milano*:** Ines Arpino, Sara Sher

***Ospedale San Paolo - University of Milano, Milano*:** Concezione Tommasino, Francesca Rapido, Paola Morelli

***University of Naples “Federico II” Naples*:** Maria Vargas, Giuseppe Servillo

***Policlinico "P. Giaccone", Palermo*:** Andrea Cortegiani, Santi Maurizio Raineri, Francesca Montalto, Vincenzo Russotto, Antonino Giarratano

***Azienda Ospedaliero-Universitaria, Parma*:** Marco Baciarello, Michela Generali, Giorgia Cerati

***Santa Maria degli Angeli, Pordenone*:** Yigal Leykin

***Ospedale Misericordia e Dolce - Usl4 Prato, Prato*:** Filippo Bressan, Vittoria Bartolini, Lucia Zamidei

***University hospital of Sassari, Sassari*:** Luca Brazzi, Corrado Liperi, Gabriele Sales, Laura Pistidda

***Insubria University, Varese*:** Paolo Severgnini, Elisa Brugnoni, Giuseppe Musella, Alessandro Bacuzzi

Republic of Kosovo

***Distric hospital Gjakova, Gjakove*:** Dalip Muhardri

***University Clinical Center of Kosova, Prishtina*:** Agreta Gecaj-Gashi, Fatos Sada

***Regional Hospital ”Prim.Dr. Daut Mustafa”, Prizren*:** Adem Bytyqi

Lithuania

***Medical University Hospital, Hospital of Lithuanian University of Health Sciences, Kaunas*:** Aurika Karbonskiene, Ruta Aukstakalniene, Zivile Teberaite, Erika Salciute

***Vilnius University Hospital - Institute of Oncology, Vilnius*:** Renatas Tikuisis, Povilas Miliauskas

***Vilnius University Hospital - Santariskiu Clinics, Vilnius*:** Sipylaite Jurate, Egle Kontrimaviciute, Gabija Tomkute

Malta

***Mater Dei Hospital, Msida*:** John Xuereb, Maureen Bezzina, Francis Joseph Borg

Netherlands

***Academic Medical Centre, University of Amsterdam*:** Sabrine Hemmes, Marcus Schultz, Markus Hollmann, Irene Wiersma, Jan Binnekade, Lieuwe Bos

***VU University Medical Center, Amsterdam*:** Christa Boer, Anne Duvekot

***MC Haaglanden, Den Haag*:** Bas in  ‘t  Veld, Alice Werger, Paul Dennesen, Charlotte Severijns

***Westfriesgasthuis, Hoorn*:** Jasper De Jong, Jens Hering, Rienk van Beek

Norway

***Haukeland University Hospital, Bergen*:** Stefan Ivars, Ib Jammer

***Førde Central Hospital /Førde Sentral Sykehus, Førde*:** Alena Breidablik

***Martina Hansens Hospital, Gjettum*:** Katharina Skirstad Hodt, Frode Fjellanger, Manuel Vico Avalos

***Bærum Hospital, Vestre Viken, Rud*:** Jannicke Mellin-Olsen, Elisabeth Andersson

***Stavanger University Hospital, Stavanger*:** Amir Shafi-Kabiri

Panama

***Hospital Santo Tomás, Panama*:** Ruby Molina, Stanley Wutai, Erick Morais

Portugal

***Hospital do Espírito Santo - Évora, E.P.E, Évora.*:** Glória Tareco, Daniel Ferreira, Joana Amaral

***Centro Hospitalar de Lisboa Central, E.P.E, Lisboa.*:** Maria de Lurdes Goncalves Castro, Susana Cadilha, Sofia Appleton

***Centro Hospitalar de Lisboa Ocidental, E.P.E. Hospital de S. Francisco Xavier, Lisboa*:** Suzana Parente, Mariana Correia, Diogo Martins

***Santarem Hospital, Santarem*:** Angela Monteirosa, Ana Ricardo, Sara Rodrigues

Romania

***Spital Orasenesc, Bolintin Vale*:** Lucian Horhota

***Clinical Emergency Hospital of Bucharest, Bucharest*:** Ioana Marina Grintescu, Liliana Mirea, Ioana Cristina Grintescu

***Elias University Emergency Hospital, Bucharest*:** Dan Corneci, Silvius Negoita, Madalina Dutu, Ioana Popescu Garotescu

***Emergency Institute of Cardiovascular Diseases Inst. ''Prof. C. C. Iliescu'', Bucharest*:** Daniela Filipescu, Alexandru Bogdan Prodan

***Fundeni Clinical institute - Anaesthesia and Intensive Care, Bucharest*:** Gabriela Droc, Ruxandra Fota, Mihai Popescu

***Fundeni Clinical institute - Intensive Care Unit, Bucharest*:** Dana Tomescu, Ana Maria Petcu, Marian Irinel Tudoroiu

***Hospital Profesor D Gerota, Bucharest*:** Alida Moise, Catalin-Traian Guran

***Constanta County Emergency Hospital, Constanta*:** Iorel Gherghina, Dan Costea, Iulia Cindea

***University Emergency County Hospital Targu Mures, Targu Mures*:** Sanda-Maria Copotoiu, Ruxandra Copotoiu, Victoria Barsan, Zsolt Tolcser, Magda Riciu, Septimiu Gheorghe Moldovan, Mihaly Veres

Russia

***Krasnoyarsk State Medical University, Krasnoyarsk*:** Alexey Gritsan, Tatyana Kapkan, Galina Gritsan, Oleg Korolkov

*Burdenko Neurosurgery Institute, Moscow*: Alexander Kulikov, Andrey Lubnin

***Moscow Regional Research Clinical Institute, Moscow*:** Alexey Ovezov, Pavel Prokoshev, Alexander Lugovoy, Natalia Anipchenko

***Municipal Clinical Hospital 7, Moscow*:** Andrey Babayants, Irina Komissarova, Karginova Zalina

***Reanimatology Research Institute n.a. Negovskij RAMS, Moscow*:** Valery Likhvantsev, Sergei Fedorov

Serbia

***Clinical Center of Vojvodina, Emergency Center, Novisad*:** Aleksandra Lazukic, Jasmina Pejakovic, Dunja Mihajlovic

Slovakia

***National Cancer Institute, Bratislava*:** Zuzana Kusnierikova, Maria Zelinkova

***F.D. Roosevelt teaching Hospital, Banská Bystrica*:** Katarina Bruncakova, Lenka Polakovicova

***Faculty Hospital Nové Zámky, Nové Zámky*:** Villiam Sobona

Slovenia

***Institute of Oncology Ljubljana, Ljubljana*:** Barbka Novak-Supe, Ana Pekle-Golez, Miroljub Jovanov, Branka Strazisar

***University Medical Centre Ljubljana, Ljubljana*:** Jasmina Markovic-Bozic, Vesna Novak-Jankovic, Minca Voje, Andriy Grynyuk, Ivan Kostadinov, Alenka Spindler-Vesel

Spain

***Hospital Sant Pau, Barcelona*:** Victoria Moral, Mari Carmen Unzueta, Carlos Puigbo, Josep Fava

***Hospital Universitari Germans Trias I Pujol, Barcelona*:** Jaume Canet, Enrique Moret, Mónica Rodriguez Nunez, Mar Sendra, Andrea Brunelli, Frederic Rodenas

***University of Navarra, Pamplona*:** Pablo Monedero, Francisco Hidalgo Martinez, Maria Jose Yepes Temino, Antonio Martínez Simon, Ana de Abajo Larriba

***Corporacion Sanitaria Parc Tauli, Sabadell*:** Alberto Lisi, Gisela Perez, Raquel Martinez

***Consorcio Hospital General Universitario de Valencia, Valencia*:** Manuel Granell, Jose Tatay Vivo, Cristina Saiz Ruiz, Jose Antonio de Andrés Ibañez

***Hospital Clinico Valencia, Valencia*:** Ernesto Pastor, Marina Soro, Carlos Ferrando, Mario Defez

***Hospital Universitario Rio Hortega, Valladolid*:** Cesar Aldecoa Alvares-Santullano, Rocio Perez, Jesus Rico

Sweden

***Central Hospital in Kristianstad*:** Monir Jawad, Yousif Saeed, Lars Gillberg

Turkey

***Ufuk University Hospital Ankara, Ankara*:** Zuleyha Kazak Bengisun, Baturay Kansu Kazbek

***Akdeniz University Hospital, Antalya*:** Nesil Coskunfirat, Neval Boztug, Suat Sanli, Murat Yilmaz, Necmiye Hadimioglu

***Istanbul University, Istanbul medical faculty, Istanbul*:** Nuzhet Mert Senturk, Emre Camci, Semra Kucukgoncu, Zerrin Sungur, Nukhet Sivrikoz

***Acibadem University, Istanbul*:** Serpil Ustalar Ozgen, Fevzi Toraman

***Maltepe University, Istanbul*:** Onur Selvi, Ozgur Senturk, Mine Yildiz

***Dokuz Eylül Universitesi Tip Fakültesi, Izmir*:** Bahar Kuvaki, Ferim Gunenc, Semih Kucukguclu, Şule Ozbilgin

***Şifa University Hospital, İzmir*:** Jale Maral, Seyda Canli

***Selcuk University faculty of medicine, Konya*:** Oguzhan Arun, Ali Saltali, Eyup Aydogan

***Fatih Sultan Mehmet Eğitim Ve Araştirma Hastanesi, Istanbul*:** Fatma Nur Akgun, Ceren Sanlikarip, Fatma Mine Karaman

Ukraine

***Institute Of Surgery And Transplantology, Kiev*:** Andriy Mazur

***Zaporizhzhia State Medical University, Zaporizhzhia*:** Sergiy Vorotyntsev

United Kingdom

**SWARM Research Collaborative: for full list of SWARM contributors please see www.ukswarm.com**

***Northern Devon Healthcare NHS Trust, Barnstaple*:** Guy Rousseau, Colin Barrett, Lucia Stancombe

***Golden Jubilee National Hospital, Clydebank, Scotland*:** Ben Shelley, Helen Scholes

***Darlington Memorial Hospital, County Durham and Darlington Foundation NHS Trust, Darlington*:** James Limb, Amir Rafi, Lisa Wayman, Jill Deane

***Royal Derby Hospital, Derby:*** David Rogerson, John Williams, Susan Yates, Elaine Rogers

***Dorset County Hospital, Dorchester*:** Mark Pulletz, Sarah Moreton, Stephanie Jones

***The Princess Alexandra NHS Hospital Trust, Essex*:** Suresh Venkatesh, Maudrian Burton, Lucy Brown, Cait Goodall

***Royal Devon and Exeter NHS Foundation Trust, Exeter*:** Matthew Rucklidge, Debbie Fuller, Maria Nadolski, Sandeep Kusre

***Hospital James Paget University Hospital NHS Foundation Trust, Great Yarmouth*:** Michael Lundberg, Lynn Everett, **Helen Nutt**

***Royal Surrey County Hospital NHS Foundation Trust, Guildford*:** Maka Zuleika, Peter Carvalho, Deborah Clements, Ben Creagh-Brown

***Kettering General Hospital NHS Foundation Trust, Kettering*:** Philip Watt, Parizade Raymode

***Barts Health NHS Trust, Royal London Hospital, London*:** Rupert Pearse, Otto Mohr, Ashok Raj, Thais Creary

***Newcastle Upon Tyne Hospitals NHS Trust The Freeman Hospital High Heaton, Newcastle upon Tyne*:** Ahmed Chishti, Andrea Bell, Charley Higham, Alistair Cain, Sarah Gibb, Stephen Mowat

***Derriford Hospital Plymouth Hospitals NHS Trust, Plymouth*:** Danielle Franklin, Claire West, Gary Minto, Nicholas Boyd

***Royal Hallamshire Hospital, Sheffield*:** Gary Mills, Emily Calton, Rachel Walker, Felicity Mackenzie, Branwen Ellison, Helen Roberts

***Mid Staffordshire NHS, Stafford*:** Moses Chikungwa, Clare Jackson

***Musgrove Park Hospital, Taunton*:** Andrew Donovan, Jayne Foot, Elizabeth Homan

***South Devon Healthcare NHS Foundation Trust /Torbay Hospital, Torquay, Torbay*:** Jane Montgomery, David Portch, Pauline Mercer, Janet Palmer

***Royal Cornwall Hospital, Truro*:** Jonathan Paddle, Anna Fouracres, Amanda Datson, Alyson Andrew, Leanne Welch

***Mid Yorkshire Hospitals NHS Trust; Pinderfields Hospital, Wakefield*:** Alastair Rose, Sandeep Varma, Karen Simeson

***Sandwell and West Birmingham NHS Trust, West Bromich:*** Mrutyunjaya Rambhatla, Jaysimha Susarla, Sudhakar Marri, Krishnan Kodaganallur, Ashok Das, Shivarajan Algarsamy, Julie Colley

***York Teaching Hospitals NHS Foundation Trust, York*:** Simon Davies, Margaret Szewczyk, Thomas Smith

United States

***University of Colorado School of Medicine/University of Colorado Hospital, Aurora*:** Ana Fernandez- Bustamante, Elizabeth Luzier, Angela Almagro

***Massachusetts General Hospital, Boston*:** Marcos Vidal Melo, Luiz Fernando, Demet Sulemanji

***Mayo Clinic, Rochester*:** Juraj Sprung, Toby Weingarten, Daryl Kor, Federica Scavonetto, Yeo Tze
