# Supplementary material for: Geo–economic variations in epidemiology, ventilation management and outcome of patients receiving intraoperative ventilation during general anesthesia– posthoc analysis of an observational study in 29 countries
Source: BMC Anesthesiol. 2022 Jan 7;22:15. doi: 10.1186/s12871-021-01560-x (PMC8740416; doi:10.1186/s12871-021-01560-x)
Supplement: Supplementary file 2 — Additional file 2. ARISAT score. The ARISCAT risk score is used to calculate the risk for developing postoperative pulmonary complications. [file 12871_2021_1560_MOESM2_ESM.docx]

| **Additional file 2.** ARISCAT risk score  *Independent predictors of risk for postoperative pulmonary complications identified in logistic regression model* | | | |
| --- | --- | --- | --- |
|  | **multivariate analysis** | **ß-coefficients** | **risk score**^†^ |
| Age (years) |  |  |  |
| ≤ 50 | 1 |  |  |
| 51 – 80 | 1.4 (0.6 - 3.3) | 0.331 | 3 |
| >80 | 5.1 (1.9 - 13.3) | 1.619 | 16 |
| Preoperative SpO_2_ (%) |  |  |  |
| ≥96 | 1 |  |  |
| 91 – 95 | 2.2 (1.2 - 4.2) | 0.802 | 8 |
| ≤90 | 10.7 (4.1 - 28.1) | 2.375 | 24 |
| Respiratory infection in the last month | 5.5 (2.6 - 11.5) | 1.698 | 17 |
| Preoperative anemia (≤ 100 g/L) | 3.0 (1.4 - 6.5) | 1.105 | 11 |
| Surgical incision |  |  |  |
| Peripheral | 1 |  |  |
| Upper abdominal | 4.4 (2.3 - 8.5) | 1.480 | 15 |
| Intrathoracic | 11.4 (4.9 - 26.0) | 2.431 | 24 |
| Duration of surgery (hours) |  |  |  |
| ≤2 | 1 |  |  |
| 2 to 3 | 4.9 (2.4 - 10.1) | 1.593 | 16 |
| >3 | 9.7 (4.7 - 19.9) | 2.268 | 23 |
| Emergency procedure | 2.2 (1.04 - 4.5) | 0.768 | 8 |
| ^†^ The simplified risk score was the sum of each logistic regression coefficient multiplied by 10, after rounding off its value. The higher the score, the greater the risk of PPC. Sum <26 = low risk, 26-44 = intermediate risk, >44 = high risk.  *CI: confidence interval; OR: odds ratio; SpO_2_: oxyhemoglobin saturation by pulse oximetry* | | | |
