# Supplementary material for: Geo–economic variations in epidemiology, ventilation management and outcome of patients receiving intraoperative ventilation during general anesthesia– posthoc analysis of an observational study in 29 countries
Source: BMC Anesthesiol. 2022 Jan 7;22:15. doi: 10.1186/s12871-021-01560-x (PMC8740416; doi:10.1186/s12871-021-01560-x)
Supplement: Supplementary file 3 — Additional file 3. Definitions of postoperative pulmonary complications. A description of the definitions of postoperative pulmonary complications used in this analysis. [file 12871_2021_1560_MOESM3_ESM.docx]

**Additional file 3.** Definitions of postoperative pulmonary complications

| **Postoperative pulmonary complication** | **Definition** |
| --- | --- |
| Respiratory failure | SpO_2_ < 90% or a PaO_2_ < 60 mm Hg with oxygen therapy, or need for non–invasive positive pressure ventilation |
| Mechanical ventilation | Need for new invasive ventilation after surgery, or unexpected prolonged invasive ventilation after discharge from the operating room |
| Acute Distress Respiratory Syndrome | Conform the current Berlin definitions, using the following criteria; presence of acute hypoxemic respiratory failure with an onset within 7 days after surgery with (new or progressive) bilateral opacities on chest X–ray or CT–scan that is not fully explained by effusion, lobar or lung collapse, or nodules. Cardiac failure should be excluded as the primary cause of acute respiratory failure. |
| Pneumonia | New or progressive lung infiltrates at the chest X–ray or CT, and at least two of the following criteria: fever > 38°C (100.4 °F), leukocytosis or leukopenia (WBC count > 12000 or < 4000 cells/mm^3^), and purulent secretion. |
| Pneumothorax | Air in the pleural space without blood presence, as confirmed by chest radiograph. |

Patients who develop at least one postoperative pulmonary complication were considered as meeting the primary endpoint.

CT–scan: Computer Tomography; WBC: White Blood Cell
