# Supplementary material for: Geo–economic variations in epidemiology, ventilation management and outcome of patients receiving intraoperative ventilation during general anesthesia– posthoc analysis of an observational study in 29 countries
Source: BMC Anesthesiol. 2022 Jan 7;22:15. doi: 10.1186/s12871-021-01560-x (PMC8740416; doi:10.1186/s12871-021-01560-x)
Supplement: Supplementary file 4 — Additional file 4. Definitions of intraoperative complications. A description of the definitions of intraoperative complications used in this analysis. [file 12871_2021_1560_MOESM4_ESM.docx]

**Additional file 4**. Definitions of intraoperative complications

| **Intraoperative complication** | **Definition** |
| --- | --- |
| Any desaturation | Oxygen saturation, SpO_2_ < 92% |
| Use of any unplanned recruitment maneuvers | Ventilation strategies aimed at restoring aeration of the lungs; recruitment maneuvers could have been performed by ‘bag squeezing’ (sustained hyperinflation using a ventilation balloon or bag), with the OR ventilator (stepwise increase in V_T_ at a constant PEEP, or a stepwise increase in PEEP at a constant V_T,_ or the use of an inspiratory hold for a fixed time) or a combination of the above. |
| Need for ventilator pressure reduction | Any change in ventilator settings to decrease an unacceptable high airway pressure during surgery |
| New onset of expiratory flow limitation | End–expiration expiratory flow higher than zero at the visual analysis of the flow curve |
| Hypotension | Systolic arterial pressure < 90 mm Hg for at least 3 minutes |
| Need for vasoactive drugs | Use of vasoactive drugs to correct for hypotension |
| Arrhythmia | Any new onset of arterial fibrillation, sustained ventricular tachycardia, supraventricular tachycardia, or ventricular fibrillation |

V_T_: tidal volume; PEEP: Positive End–Expiratory Pressure
