# Supplementary material for: Geo–economic variations in epidemiology, ventilation management and outcome of patients receiving intraoperative ventilation during general anesthesia– posthoc analysis of an observational study in 29 countries
Source: BMC Anesthesiol. 2022 Jan 7;22:15. doi: 10.1186/s12871-021-01560-x (PMC8740416; doi:10.1186/s12871-021-01560-x)
Supplement: Supplementary file 5 — Additional file 5. CONSORT flow chart of the study population. Flowchart with information on how the study population was obtained. [file 12871_2021_1560_MOESM5_ESM.docx]

**Additional file 5.** CONSORT flowchart
