# Supplementary material for: Geo–economic variations in epidemiology, ventilation management and outcome of patients receiving intraoperative ventilation during general anesthesia– posthoc analysis of an observational study in 29 countries
Source: BMC Anesthesiol. 2022 Jan 7;22:15. doi: 10.1186/s12871-021-01560-x (PMC8740416; doi:10.1186/s12871-021-01560-x)
Supplement: Supplementary file 6 — Additional file 6. Posthoc pairwise analysis for numerical data. Posthoc Dunn’s test for pairwise multiple comparison of the ranked data. [file 12871_2021_1560_MOESM6_ESM.docx]

|  | **Additional file 6.** Posthoc test for numerical data | | | |
| --- | --- | --- | --- | --- |
|  | | **Z-value** | **Estimated median difference** | **Adjusted *P*-value** |
| **Age** | |  |  |  |
| HIC – LMIC | | 5.177 | 5.0 years | <0.001 |
| HIC – UMIC | | 9.182 | 5.0 years | <0.001 |
| UMIC – LMIC | | 0.584 | <0.001 years | 1.000 |
| **Height** | |  |  |  |
| HIC – LMIC | | -2.303 | 2 cm | 0.064 |
| HIC – UMIC | | 1.641 | <0.001 cm | 0.303 |
| UMIC – LMIC | | 2.836 | 2 cm | 0.014 |
| **ARISCAT** | |  |  |  |
| HIC – LMIC | | -3.245 | 2 points | 0.004 |
| HIC – UMIC | | -1.137 | <0.001 points | 0.767 |
| UMIC – LMIC | | 2.270 | <0.001 points | 0.070 |
| **Preoperative SpO_2_** | |  |  |  |
| HIC – LMIC | | -2.781 | <0.001% | 0.016 |
| HIC – UMIC | | -5.641 | <0.001% | <0.001 |
| UMIC – LMIC | | -0.642 | <0.001% | 1.000 |
| **Intraoperative RR** | |  |  |  |
| HIC – LMIC | | -2.818 | <0.001 rpm | 0.015 |
| HIC – UMIC | | -3.616 | <0.001 rpm | <0.001 |
| UMIC – LMIC | | 0.457 | <0.001 rpm | 1.000 |

Posthoc Dunn’s test for pairwise multiple comparison of the ranked data. Bonferroni method used to adjust the documented p-values.

*HIC: high–income countries; UMIC: upper middle–income countries; LMIC: lower middle–income countries; RR: Respiratory Rate.*
