# Supplementary material for: Geo–economic variations in epidemiology, ventilation management and outcome of patients receiving intraoperative ventilation during general anesthesia– posthoc analysis of an observational study in 29 countries
Source: BMC Anesthesiol. 2022 Jan 7;22:15. doi: 10.1186/s12871-021-01560-x (PMC8740416; doi:10.1186/s12871-021-01560-x)
Supplement: Supplementary file 7 — Additional file 7. Posthoc pairwise analysis for categorical data. Chi-square test for pairwise comparison. [file 12871_2021_1560_MOESM7_ESM.docx]

|  | **Additional 7.** Posthoc test for categorical data | | |
| --- | --- | --- | --- |
|  | | **Cramér’s V** | **Adjusted *P*-value** |
| **ASA** | |  |  |
| HIC – LMIC | | 0.014 | 0.192 |
| HIC – UMIC | | 0.026 | 0.011* |
| UMIC – LMIC | | 0.005 | 0.856 |
| **Smoker** | |  |  |
| HIC – LMIC | | 0.001 | 0.905 |
| HIC – UMIC | | 0.032 | 0.002* |
| UMIC – LMIC | | 0.038 | 0.145 |
| **Urgency of surgery** | |  |  |
| HIC – LMIC | | 0.090 | <0.001* |
| HIC – UMIC | | 0.103 | <0.001* |
| UMIC – LMIC | | 0.085 | 0.005* |
| **Epidural** | |  |  |
| HIC – LMIC | | 0.032 | 0.003* |
| HIC – UMIC | | 0.034 | 0.001* |
| UMIC – LMIC | | 0.027 | 0.292 |
| **Muscle paralyzing agents** | |  |  |
| HIC – LMIC | | 0.043 | <0.001* |
| HIC – UMIC | | 0.038 | <0.001* |
| UMIC – LMIC | | 0.044 | 0.085 |
| **Neuromuscular blockade reversal agent** | |  |  |
| HIC – LMIC | | 0.084 | <0.001* |
| HIC – UMIC | | 0.052 | <0.001* |
| UMIC – LMIC | | 0.104 | <0.001* |
| **Neuromuscular monitoring** | |  |  |
| HIC – LMIC | | 0.039 | <0.001* |
| HIC – UMIC | | 0.128 | <0.001* |
| UMIC – LMIC | | 0.143 | <0.001* |
| **Need for a blood transfusion** | |  |  |
| HIC – LMIC | | 0.009 | 0.417 |
| HIC – UMIC | | 0.033 | 0.001* |
| UMIC – LMIC | | 0.019 | 0.468 |

Chi-square test for pairwise comparison. *To adjust for multiple testing, in this table a P-value of <0.017 is considered as significant.

*HIC: high–income countries; UMIC: upper middle–income countries; LMIC: lower middle–income countries*
