# Supplementary material for: Geo–economic variations in epidemiology, ventilation management and outcome of patients receiving intraoperative ventilation during general anesthesia– posthoc analysis of an observational study in 29 countries
Source: BMC Anesthesiol. 2022 Jan 7;22:15. doi: 10.1186/s12871-021-01560-x (PMC8740416; doi:10.1186/s12871-021-01560-x)
Supplement: Supplementary file 8 — Additional file 8. Multivariate model of factors associated with the development of PPC. HIC and UMIC were compared to LMIC and centers were entered as random effect. [file 12871_2021_1560_MOESM8_ESM.docx]

**Additional file 8**. Multivariate logistic model of factors associated with the development of PPC compared to LMIC and centers as random effect.

|  | **Odds ratio (95% CI)** | **p-value** |
| --- | --- | --- |
| **Upper middle income countries** | **0.054 (0.026 to 0.110)** | **<0.001** |
| **High income countries** | **0.035 (0.020 to 0.062)** | **<0.001** |
| ARISCAT | 1.394 (1.158 to 1.675) | <0.001 |
| ASA ≥3 | 2.350 (1.615 to 3.416) | <0.001 |
| Smoker | 0.898 (0.586 to 1.343) | 0.610 |
| *Urgent surgery | 2.740 (1.736 to 4.229) | <0.001 |
| *Emergency surgery | 3.409 (1.587 to 6.920) | 0.001 |
| Intraoperative need for vasopressors | 1.739 (1.177 to 2.562) | 0.005 |
| Intraoperative need for transfusion | 2.738 (1.581 to 4.617) | <0.001 |
| Type of surgery |  |  |
| Lower gastrointestinal | 1.538 (0.957 to 2.427) | 0.069 |
| Upper gastrointestinal | 2.036 (1.353 to 3.025) | <0.001 |
| Aortic | 1.229 (0.189 to 4.511) | 0.789 |
| Mechanical ventilation |  |  |
| Peak pressure, cm H_2_O | 1.249 (0.947 to 1.629) | 0.108 |
| Tidal volume per PBW, mL | 0.865 (0.712 to 1.049) | 0.144 |
| Driving pressure, cm H_2_O | 0.943 (0.727 to 1.226) | 0.658 |
| PEEP, cm H_2_O | 1.069 (0.855 to 1.342) | 0.560 |

*Urgency of surgery: elective: surgery that is scheduled in advance because it does not involve a medical emergency; urgent: surgery required within <48 hours; emergency: non-elective surgery performed when the patient’s life or wellbeing is in direct jeopardy.

*ARISCAT: Assess Respiratory Risk in Surgical Patients in Catalonia; ASA: American Society of Anesthesiology; PBW: Predicted BodyWeight; PEEP: Positive-end-expiratory Pressure;*
